# Kinetic variations between reverse transcriptases of viral protein X coding and noncoding lentiviruses

**DOI:** 10.1186/s12977-014-0111-y

**Published:** 2014-12-19

**Authors:** Gina M Lenzi, Robert A Domaoal, Dong-Hyun Kim, Raymond F Schinazi, Baek Kim

**Affiliations:** Center for Drug Discovery, Center for AIDS Research, Department of Pediatrics, Emory University School of Medicine, 1760 Haygood Drive, Atlanta, GA USA; College of Pharmacy, Kyung-Hee University, Seoul, South Korea; Veterans Affairs Medical Center, Decatur, GA USA

**Keywords:** Lentivirus, Reverse transcriptase, Enzyme kinetics, Vpx, SAMHD1, dNTPs, Macrophages

## Abstract

**Background:**

Host SAM domain and HD domain-containing protein 1 (SAMHD1) suppresses reverse transcription kinetics of HIV-1 in nondividing cells such as macrophages by hydrolyzing and nearly depleting cellular dNTPs, which are the substrates of viral reverse transcriptase (RT). However, unlike HIV-1, HIV-2 and SIVsm encode viral protein X (Vpx), which counteracts the dNTPase activity of SAMHD1 and elevates dNTP concentration, allowing the viruses to replicate under abundant dNTP conditions even in nondividing cells.

**Findings:**

Here we tested whether RTs of these Vpx coding and noncoding lentiviruses display different enzyme kinetic profiles in response to dNTP concentrations. For this test, we characterized an extensive collection of RTs from 7 HIV-1 strains, 4 HIV-2 strains and 7 SIV strains, and determined their steady-state kinetic parameters. The *K*_*m*_ values of all HIV-1 RTs were consistently low and close to the low dNTP concentrations found in macrophages. However, the *K*_*m*_ values of SIV and HIV-2 RTs were not only higher than those of HIV-1 RTs but also varied significantly, indicating that HIV-2/SIV RTs require higher dNTP concentrations for efficient DNA synthesis, compared to HIV-1 RT. However, the *k*_*cat*_ values of all eighteen lentiviral RTs were very similar.

**Conclusions:**

Our biochemical analysis supports the hypothesis that the enzymological properties, particularly, *K*_*m*_ values, of lentivirus RTs, are mechanistically tied with the cellular dNTP availability in nondividing target cells, which is controlled by SAMHD1 and Vpx.

## Findings

Lentiviruses such as HIV-1, HIV-2 and SIV infect both activated/dividing CD4^+^ T cells and various nondividing myeloid cell types including macrophages and microglia during the course of their pathogenesis [1,2]. However, the kinetics of HIV-1 replication in these nondividing cells is significantly delayed, compared to activated CD4^+^ T cells [2,3]. Nondividing cells maintain lower dNTP concentrations than dividing cells that can activate cellular dNTP biosynthesis at S phase [4]. Thus due to the limited dNTP availability, nondividing macrophages are suboptimal for supporting proviral DNA synthesis of lentiviruses as compared to the activated and constantly dividing CD4^+^ T cells [2,5]. Indeed, cellular dNTP concentrations are ~200 times lower in macrophage (20–40 nM) than activated CD4^+^ T cells (1–16 μM) [2]. A series of recent studies showed that the host SAM domain and HD domain-containing protein 1 (SAMHD1) protein has dNTP hydrolase and RNase activities and serves as a restriction factor that can delay the replication kinetics of lentiviruses [6-10], and the dNTP hydrolase activity of SAMHD1 is responsible for the poor dNTP availability in the viral nondividing target cell types such as macrophages and dendritic cells (DCs) [11].

Interestingly, unlike HIV-1, HIV-2 replicates more rapidly in nondividing cells [12]. This phenotype is directly linked to a viral accessory protein, called viral protein X (Vpx), which is encoded by HIV-2 and many SIV strains [13,14]. Recent studies revealed that Vpx targets SAMHD1 for proteasomal degradation through the E3 ubiquitination pathway [15,16], and the cellular depletion of SAMHD1 leads to elevated dNTP concentrations and accelerated reverse transcription in macrophages, resting CD4^+^ T cells, and DCs [17]. However, unlike HIV-2/SIV which rapidly replicate under high dNTP concentration conditions even in the nondividing cells, the proviral DNA synthesis of HIV-1 lacking Vpx is kinetically restricted in the nondividing target cell types due to the limited dNTP pools established by SAMHD1 [18].

Since Vpx-lacking HIV-1 replicates at extremely low dNTP concentration environments in macrophages, we tested whether RTs of HIV-1 strains display a higher affinity for dNTPs and lower *K*_*m*_ values close to the dNTP concentrations found in macrophages as compared with RTs of Vpx-encoding lentiviruses such as HIV-2 and SIV where the selective pressure to function optimally at low dNTP concentrations is lifted by Vpx.

To test this, we cloned, overexpressed and purified RT proteins from 7 HIV-1 strains of various subtypes (A, B, C, D, F/H), 4 strains of HIV-2 and 7 strains of SIV [19]. The NIH AIDS Reagent Program and collaborators (Drs. V.M. Hirsch and J. Overbaugh) generously offered the near-full length molecular clones for the different HIV-1, HIV-2 and SIV strains. Briefly, the RT genes were cloned from these molecular clones into pET28a creating an N-terminus six histidine tag with NdeI/ XhoI sites and then overexpressed in *E. coli* BL21 (Novagen, WI) with 1 mM IPTG, lysed with sonication, and purified using a HisBind Purification Kit (EMD Millipore) to greater than 95% purity. Protein yields were typically 8–12 mg/ L bacterial culture and 1–3 protein preps were used for each experiment.

**Figure 1:**
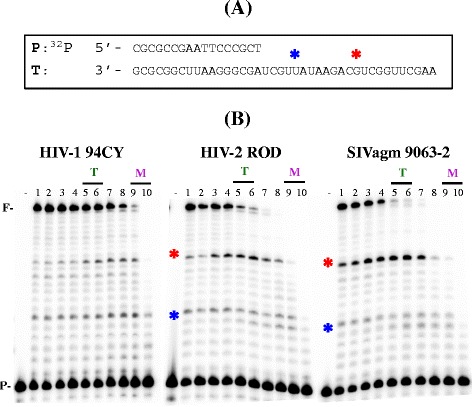
**Effect of dNTP concentration on RNA-dependent DNA polymerization activity for lentiviral RT proteins. (A)** 5’ ^32^P-labeled 17-mer primer (P) annealed to 40-mer RNA template. **(B)** The T/P was extended by 18 purified RT proteins under the condition described in Experimental Procedures at different dNTP concentrations (lanes 1–10: 50 μM, 25 μM, 10 μM, 5 μM, 1 μM, 500 nM, 250 nM, 100, nM, 50 nM, 25 nM). HIV-1 strains used were HXB2, NL4-3, 94CY, 92RW, 93IN, 94UG, and 93BR. HIV-2 strains used were Ghana1, ST1, ROD, and ROD10. SIV stains used were Mac239, Mne CL8, Mne 170, Agm155-4, Agm Gri-1, Agm 9063–2, and Agm Tan-1. RT activity used in this assay generated approximately 50% primer extension as determined by 40 bp fully extended product (F) at the highest dNTP concentration (lane 1). Among 18 RT proteins, the reactions with HIV-1 94CY, HIV-2 ROD, and SIVagm 9063–2 are shown in this figure, “*” indicates pause sites produced by kinetic delays of dNTP incorporations at lower dNTP concentrations. (−) no RT control. T: dNTP concentrations found in activated CD4+ T cells, M: dNTP concentrations found in macrophages.

First we examined the effect of dNTP concentration on RNA-dependent DNA polymerization activity of these purified RT enzymes using a 40-mer RNA template (T) annealed to a 5’-^32^P labeled 17-mer DNA primer (P, Figure 1A) at dNTP concentrations observed in activated/dividing CD4^+^ T cells (“T” in Figure 1B) and nondividing macrophages (“M” in Figure 1B) in a primer extension assay previously described [20]. As shown in representative gels with RTs from HIV-1, HIV-2 and SIV groups (Figure 1B, other RT data not shown), upon the use of an equal DNA polymerase activity measured at the highest dNTP concentration (lane 1, 50 μM), all HIV-1 RTs (i.e. HIV-1 94CY in Figure 1B) were able to extend the primer efficiently even at low dNTP concentrations found in macrophages (“M”), while many HIV-2 and SIV RTs (i.e. HIV-2 ROD and SIVagm 9063–2 in Figure 1B) failed to fully extend products at the low dNTP concentrations found in macrophages. Significant pause sites (see “*” in Figure 1B), which are generated by the kinetic delay of dNTP incorporation, are more evident in HIV-2 and SIV RTs, compared to HIV-1 RTs. This initial qualitative analysis shown in Figure 1 suggests that RT proteins from the three groups of lentiviruses (HIV-1, HIV-2 and SIV) have different dNTP concentration dependent DNA polymerase activity profiles.

**Figure 2:**
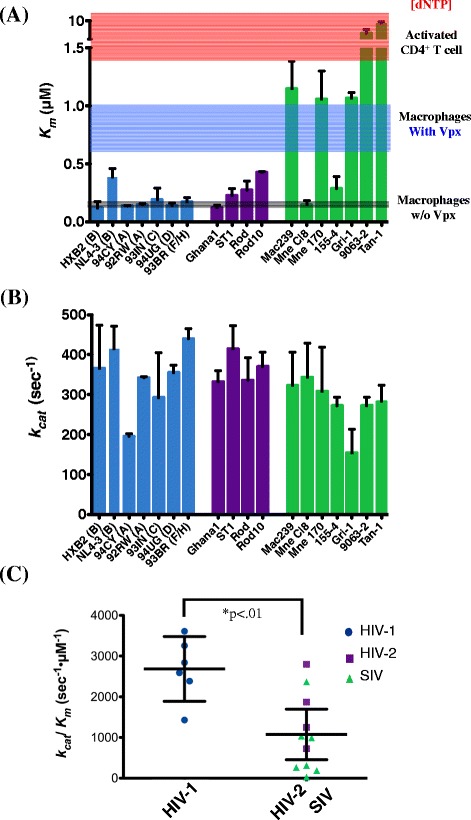
**Comparison of the steady-state kinetic parameters for 18 lentiviral RT proteins.** The *K*_*m*_ **(A)** and *k*_*cat*_ **(B)** values of the 18 different RT enzymes (blue bars, HIV-1 RTs; purple bars, HIV-2 RTs; green bars, SIV RTs) were determined from the reactions described in Figure 1. dNTP concentrations found in macrophages (grey), activated CD4+ T cells (pink), and macrophages exposed to Vpx (blue) were marked in **(A)** [17]. **(C)** The overall catalytic efficiency values (*k*_*cat*_ / *K*_*m*_) were plotted with a 95% confidence interval and the efficiency difference between RTs of Vpx coding and noncoding viruses were compared. The *V*_*max*_ and *K*_*m*_ values were determined by fitting the data to the Michaelis-Menten equation using nonlinear regression with Kaleidagraph (Synergy Software). *k*_*cat*_ was determined by dividing *V*_*max*_ by molar enzyme concentration. Values reported represent means and standard deviations of HIV-1 and the group HIV-2/ SIV. Two-tailed Student’s t tests were used for the two group comparisons (Vpx + vs Vpx: p < 0.01; HIV-1 vs SIV: p < 0.01; HIV-1 vs HIV-2: p < 0.1; HIV-2 vs SIV: p = 0.12).

Next, in order to quantitatively and mechanistically differentiate the RT activity discrepancy among the 18 RT proteins, we determined their steady-state *K*_*m*_ and *k*_*cat*_ values using the reaction conditions described in Figure 1. As summarized in Figure 2A, we found that the average *K*_*m*_ value for RTs from HIV-1 strains tested was 10 fold lower than those from HIV-2 and SIV combined (0.179 vs. 1.79 μM). This suggests that most Vpx encoding HIV-2 and SIV have RTs that require higher concentrations of dNTPs to reach half maximal velocity as compared with RTs from HIV-1 strains. Of note, it has been previously reported that Vpx from some HIV-2 and SIV strains fails to efficiently degrade the host SAMHD1 which may explain the low *K*_*m*_ values for a few of the Vpx encoding strains [21,22].

We found that the there was no statistically significant difference in catalytic turnover (*k*_*cat*_) among the 18 RT proteins tested (Figure 2B). This suggests that the turnover of substrate per enzyme is well conserved and unaffected within lentiviruses regardless of Vpx. Next we compared the overall steady-state catalytic efficiency (*k*_*cat*_/ *K*_*m*_) of these RT enzymes. Given that the *k*_*cat*_ values were nearly identical for all RTs tested and the *K*_*m*_ values were 10 times lower for HIV-1, it was evident that the catalytic efficiencies of HIV-1 RTs were significantly higher than RTs from HIV-2 and SIV which express Vpx (Figure 2C). This suggests that RTs from HIV-1 strains are more capable of synthesizing proviral DNA than RTs from HIV-2 or SIV particularly at low dNTP concentrations.

**Figure 3:**
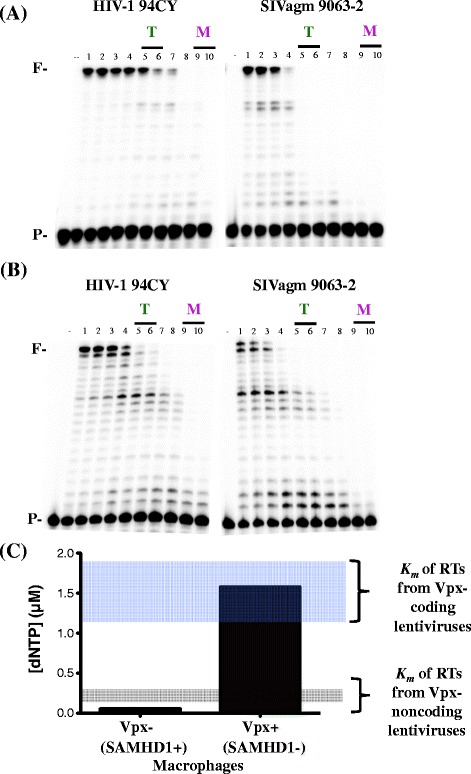
**Effect of dNTP concentration on DNA-dependent DNA polymerization activity for lentiviral RT proteins.** The primer extension reactions were conducted with the RT enzymes described except **(A)** 40-mer DNA template encoding the same sequence as the RNA template used in Figure 1 and **(B)** 48-mer DNA template encoding conserved HIV-1 PBS under the same reaction condition described in Figure 1. **(C)** Scheme explaining potential mechanistic ties between *K*_*m*_ values of RT enzymes from lentiviruses encoding or non-encoding Vpx and cellular dNTP pools modulated by SAMHD1 and Vpx in macrophages.

Next, we tested whether the observations shown in Figures 1 and 2 with RNA-dependent DNA polymerase activity of the RT enzyme are also common in their DNA-dependent DNA polymerase activity by employing a DNA template encoding the same sequence as the RNA template used in Figures 1 and 2. As shown in Figure 3A, HIV-1 94CY RT continues to extend at low dNTP concentrations as compared with SIVagm 9063–2 RT. Finally, we also tested whether the same discrepancy between HIV-1 RTs and other RTs can be observed in a template encoding a viral sequence, the primer binding site (PBS), which is one of the most conserved viral sequences among lentiviruses. As shown in Figure 3B, again, HIV-1 94CY RT enzymes are more capable of extending the primer, compared to SIVagm 9063–2 RT enzymes at the low macrophage dNTP concentrations. Therefore, the data shown in Figures 3A and 3B support that HIV-1 RTs are more efficient than Vpx-encoding lentivirus RT enzymes regardless of the types and sequences of template.

Terminally differentiated macrophages, which permanently lack chromosomal DNA replication, harbor extremely low dNTP concentrations [2], and host SAMHD1 protein, which is a dNTPase expressed at high levels specifically in nondividing cells, contributes to the dearth of dNTPs in macrophages [11]. Therefore, viruses that replicate and synthesize DNA in macrophages encounter the selective pressure generated from low dNTP availability during viral replication. We previously reported that HIV-1 RT has a uniquely low *K*_*m*_ value for dNTP substrates compared to RTs of other retroviruses that exclusively infect dividing cells such as oncoretroviruses (i.e. MuLV RT) [23,24]. It was postulated that this low *K*_*m*_ value and the ability to efficiently synthesize DNA at low dNTP concentrations could be an evolutionary outcome of the selective pressure of low dNTP concentration found in macrophages. However, other lentiviruses such as HIV-2 and many SIV strains overcome the low dNTP selective pressure by using another mechanism: they encode Vpx that counteracts the SAMHD1 mediated low dNTP availability by elevating dNTP levels and enables these lentiviruses to replicate in high dNTP environments in the nondividing target cell types [13].

Indeed, when we conducted the most extensive enzyme kinetic analysis ever reported with 18 lentiviral RT proteins, the data show that the *K*_*m*_ values of the Vpx containing lentivirus RTs, particularly SIV RTs, significantly vary, unlike the *K*_*m*_ values of HIV-1 RT enzymes, which are consistently low and close to the low dNTP concentration found in nondividing cells. As illustrated in Figure 3C, lentiviruses expressing Vpx, which counteracts the role of SAMHD1 providing a high dNTP environment and removing the selective pressure, may have higher *K*_*m*_ values because they replicate in environments with higher substrate concentrations. Moreover, the catalytic efficiency of RTs from HIV-1 lacking Vpx is significantly increased compared with RTs of HIV-2 or SIV coding for Vpx. Overall, this extensive enzymological study with a total of 18 lentivirus RT enzymes supports a close mechanistic tie between lentivirus RT kinetics and cellular dNTP availability which is regulated by the Vpx-SAMHD1 network in nondividing viral target cells. Future studies will attempt to identify key residues by sequence alignment that contribute to these differences in steady-state kinetics.

## Abbreviations

SAMHD1: SAM domain and HD domain-containing protein 1
RT: Reverse transcriptase
Vpx: Viral protein X
DC: Dendritic cells
MuLV: Murine leukemia virus
FeLV: Feline leukemia virus
PBS: Primer binding site
